# Economic Inequality in Presenting Vision in Shahroud, Iran: Two Decomposition Methods

**DOI:** 10.15171/ijhpm.2017.48

**Published:** 2017-04-22

**Authors:** Asieh Mansouri, Mohammad Hassan Emamian, Hojjat Zeraati, Hasan Hashemi, Akbar Fotouhi

**Affiliations:** ^1^ Department of Epidemiology and Biostatistics, School of Public Health, Tehran University of Medical Sciences, Tehran, Iran.; ^2^ Ophthalmic Epidemiology Research Center, Shahroud University of Medical Sciences, Shahroud, Iran.; ^3^ Noor Ophthalmology Research Center, Noor Eye Hospital, Tehran, Iran.

**Keywords:** Blinder-Oaxaca Decomposition, Concentration Index, Inequality, Iran, Presenting Visual Acuity

## Abstract

**Background:** Visual acuity, like many other health-related problems, does not have an equal distribution in terms of socio-economic factors. We conducted this study to estimate and decompose economic inequality in presenting visual acuity using two methods and to compare their results in a population aged 40-64 years in Shahroud, Iran.

**Methods:** The data of 5188 participants in the first phase of the Shahroud Cohort Eye Study, performed in 2009, were used for this study. Our outcome variable was presenting vision acuity (PVA) that was measured using LogMAR (logarithm of the minimum angle of resolution). The living standard variable used for estimation of inequality was the economic status and was constructed by principal component analysis on home assets. Inequality indices were concentration index and the gap between low and high economic groups. We decomposed these indices by the concentration index and BlinderOaxaca decomposition approaches respectively and compared the results.

**Results:** The concentration index of PVA was -0.245 (95% CI: -0.278, -0.212). The PVA gap between groups with a high and low economic status was 0.0705 and was in favor of the high economic group. Education, economic status, and age were the most important contributors of inequality in both concentration index and Blinder-Oaxaca decomposition. Percent contribution of these three factors in the concentration index and Blinder-Oaxaca decomposition was 41.1% vs. 43.4%, 25.4% vs. 19.1% and 15.2% vs. 16.2%, respectively. Other factors including gender, marital status, employment status and diabetes had minor contributions.

**Conclusion:** This study showed that individuals with poorer visual acuity were more concentrated among people with a lower economic status. The main contributors of this inequality were similar in concentration index and Blinder-Oaxaca decomposition. So, it can be concluded that setting appropriate interventions to promote the literacy and income level in people with low economic status, formulating policies to address economic problems in the elderly, and paying more attention to their vision problems can help to alleviate economic inequality in visual acuity.

## Background

Unjust disparities in health among social subgroups of the population are defined as health inequity which is correlated with many social concepts such as poverty, lack of access to services or goods, discrimination, etc. Health inequity as a normative concept is not precisely measurable, but health inequality as a metric for indirect health inequity assessment is defined as observable, measurable, and monitorable differences between population subgroups.^1^ Inequality in health is evaluated in order to help to reduce unfair discrepancies in health.^2^

Visual impairment (VI) is a global public health problem^3^ that has obvious effects on the quality of life.^4^ There are 285 million visually impaired people in the world, 39 million with blindness and 246 million with low vision.^5^ The burden of VI is disproportionately clustered in developing countries.^6^ About 8.2% of the visually impaired people in the world are from the Eastern Mediterranean region. The corresponding percentage in African, Americas, European, South-east Asian (India excluded), and Western Paciﬁc (China excluded) regions, India and China is 9.2%, 9.3%, 9.9%, 9.8% and 5.2%, 21.9% and 26.5% respectively.^5^ The prevalence of visual acuity in the Iranian population ranges from 1.39% in Tehran^3^ to 6.81% in Sistan-va-Baluchestan.^7^ In addition to visual factors such as uncorrected refractive errors or cataracts, many socio-economic factors such as literacy or access to care can affect visual acuity.^4^ As many other health-related problems, visual acuity does not have an equal distribution in terms of socio-economic factors. According to a study in China, VI does not have an equal gender and educational distribution in such a way that its prevalence is higher in females and illiterate people as compared to their counterparts.^8^ Emamian et al^9^ found a pro-rich economic inequality in VI in Shahroud. Vision loss and blindness have an unequal distribution in terms of age, gender, race, ethnicity, socio-economic status, and geographic location, as well.^10,11^ Zheng et al^12^ concluded that visual disorders were more prevalent in illiterate and low educated people. They considered education promotion interventions as an effective way in decreasing these inequalities. According to another study in the United States, the prevalence of vision disorders such as age-related macular degeneration, cataract, diabetic retinopathy, and glaucoma does not have an equal distribution in terms of race, ethnicity, education, and economic status, and these inequalities have continued to exist during the past decade.^13^

Combes et al^14^ reported that socio-economic inequalities in mortalities/morbidities were well documented in multiple studies. Therefore, recent research in this area should be concentrated on analytical methods to explain socio-economic health inequalities. This can be done via decomposing health inequality to its contributing factors in order to explain the health outcome distribution by a set of factors that vary systematically in different socio-economic situations.^2^ Two popular inequality decomposition methods are the concentration index decomposition and Blinder-Oaxaca decomposition. Since these methods are different in the used inequality indicators and decomposition approach, we can compare their results and observe their similarities in explaining inequality via the contributors by applying both methods simultaneously for an outcome. To the best of our knowledge, none of the published data on inequality decomposition have compared these methods. Therefore, we aimed to estimate and decompose economic inequality in presenting vision acuity (PVA) with these two methods and compare some of their methodological issues and results in this study. Although we estimated and decomposed economic inequality in some eye disorders in our previous studies, we focused on only one decomposition method in all of them. In the present study, in addition to practical comparison of two very widely used methods in the decomposition of inequality, we also presented some applied aspects of them which can be a useful guide for future studies.

## Methods

The data for this study were obtained from Shahroud Eye Cohort Study (ShECS). It started in 2009 for various purposes (eg, estimation of the prevalence and incidence of major visual disorders, assessment of their prognosis, evaluation of the secular changes in major eye diseases, etc). The study population was people aged 40-64 years living in the city of Shahroud, northeast of Iran in 2009. Multistage cluster random sampling was used to select the participants. In the first phase of the study, among 6311 persons who were invited, 5190 (82.2%) participated in the study. More details about the methods used in this study have been already reported.^15^

The outcome variable in the present study was PVA (corrected or uncorrected visual acuity with which the individual habitually lives). It was measured using standard logarithm of the minimum angle of resolution (LogMAR) charts by trained staff and optometrists. A higher score on this scale indicates a poorer visual acuity. The score of the better eye was used as the outcome variable in order to include the status of both eyes in the study. We used a continuous variable (presenting vision score) instead of a binary variable (VI) for 2 reasons: (1) It enabled us to use all information on presenting vison rather than the presence or absence of visual impaired and (2) It enabled us to apply linear regression that is the best model for inequality decomposition according to Wagstaff et al.^16^

### Economic Status Variable

To measure inequality, we need a living standard variable. The living standard variable in this study was the economic status. It was measured according to McKenzie.^17^ We collected the data of some household assets. Then, possession or non-possession of the household assets was converted to a continuous variable as our economic status variable via principal component analysis (PCA). Prior to PCA, we checked the‏ suitability of the asset variables for PCA via the Kaiser-Meyer-Olkin (KMO) measure of sampling adequacy and Bartlett test of sphericity. According to Williams et al^18^ a KMO index more than 0.5 and a significant Bartlett test is necessary for PCA. In our study, the KMO index was 0.735 and Bartlett test of sphericity was significant (χ^2^= 9997.70, *P *< .001). So, PCA was allowed. In this analysis, 3 components with Eigenvalue>1 covered 51.77% of the observed variance. Considering the fact first principal component explains the highest value of the variance^1,9^ and provides reasonable estimates of wealth level effects,^17^ we constructed an economic variable using this component; in other words, the economic score for each individual was constructed via summing the asset variables weighted by the first component according to the following formula^2^:

$$A_{i}=\sum_{k}\left[f_{k}\frac{\left(a_{ik}-{\bar{a}}_{k}\right)}{s_{k}}\right]$$

where *A*_i_*, a*_ik_*, S*_k_ and *f*_k_ are asset index for individual *i*, the value of asset *k* for individual *i*, the sample mean, sample standard deviation, and the weights associated with the ﬁrst principal component, respectively.

Other variables in this study were age (years), gender, education (number of education years successfully passed), marital status (single, married, widowed, and divorced), employment status (employed/unemployed), diabetes (yes/no), hypertension (yes/no), body mass index in kg/m^2^, cigarette smoking (yes/no), and having a type of medical insurance (yes/no).‏

### Statistical Analysis

The Stata software version 11/SE was used for analysis. As participants were selected via cluster sampling, the cluster sampling effect was considered in the analysis. Shahroud is covered by nine health care centers. Each health care center was used as a stratum and the number of clusters proportionate to the population served by each center was calculated. Each cluster included at least 20 people aged 40-64 years old. More details about sampling are presented elsewhere.^15^

Economic inequality in PVA was measured using two different indices:

(*i*) Concentration index: according to Kakwani,^20,21^ it can be computed as twice the covariance of the health-related outcome variable and fractional rank in living standard distribution divided by the mean health-related outcome as shown in equation 1. In this formula *h*, *r* and* µ* are the health status of the *i*th individual, fractional rank of the *i*th individual related to the living standard variable (economic status in this study), and the average of the outcome variable, respectively.

(1) $$C=\frac{2}{\mu }\mathrm{cov}\left(h_{},r_{}\right)$$

(*ii*) Gap in the average of the outcome variable between two groups: For this index, the sample is divided into two groups by the living standard variable. A standard cut-off point for this division is the median of the living standard variable.^22^ Then, the difference between the mean or percentage of the outcome variable between the two groups is calculated.

### Inequality Decompositions

Economic inequality in PVA was decomposed by two methods: concentration index decomposition and Blinder-Oaxaca decomposition. Since these decomposition methods are regression-based, the determinants of the health variable should be first identified via a proper regression model (linear regression in this study as presented in equation 2).

(2) $$Y_{i}=a+\sum_{k}\beta_{k}x_{k}+\varepsilon_{i}$$

where *Y*_i_*, β*_k_ and* ε*_i _are outcome variable, regression coefficients, and error term, respectively.

### Concentration Index Decomposition

We decomposed concentration index according to equation 3 introduced by Wagstaff et al^16^:

(3) $$\begin{array}{l} C=\sum_{k}\left(\frac{\beta_{k}\bar{x_{k}}}{\mu }\right)C_{k}+\frac{GC_{\varepsilon }}{\mu }\\ =C_{y}+\frac{GC_{\varepsilon }}{\mu } \end{array}$$

In equation (3), *C*_k_ and *GC*_ε_ are the mean of the *k*th determinant, concentration index for the *k*th determinant (defined analogously to concentration index for the health variable in question), and generalized concentration index for *ε*_i_*,* respectively. Other parts of this equation have been already introduced. As shown, this equation is made up of two components. The first (*C*_y_) is called the deterministic component and the second is called the residual component. In the deterministic component, the part in parentheses is called elasticity, which is a unit-free measure of association and shows the amount of change in the dependent variable per unit change in the determinant.

The product of elasticity and concentration index for every determinant produces the absolute contribution of that determinant. The percentage contribution of every determinant is obtained from dividing its absolute contribution by the concentration index of the dependent variable.

The sum of the absolute contributions of all determinants produces the deterministic component. So, the residual component can be obtained via subtracting the deterministic component from the total concentration index.

### Blinder-Oaxaca Decomposition

Blinder-Oaxaca decomposition was introduced by Blinder and Oaxaca^23,24^ in 1973. They identified influential factors on the labor market discrimination using this method. In short, it decomposes the gap in outcome between two groups (eg, poor/non-poor or advantaged/disadvantaged). The aim is to determine how much of the overall gap in the outcome between the two groups is attributable to a) differences in the outcome determinants (so-called endowment or explained component) and b) differences in the determinants’ effects (so-called coefficient or unexplained component) between two groups.^2^

Therefore, in Blinder-Oaxaca decomposition, the individuals are first divided into two groups by a cut-off point of the living standard variable. Then, the outcome determinants are determined via a proper regression model (linear regression in simplest case):

In equations 4 and 5, * β* and* ε* are regression coefficients and error term, respectively.

(4) $$Y_{nonpoor}=\beta X_{nonpoor}+\varepsilon_{nonpoor}$$

(5) $$Y_{poor}=\beta X_{poor}+\varepsilon_{poor}$$

The next step is to estimate the mean of each determinant. Then, these means and *βs* are used for decomposition of the gap using equations 6:

(6) $${\bar{Y}}_{nonpoor}-{\bar{Y}}_{poor}=\left({\bar{X}}_{nonpoor}-{\bar{X}}_{poor}\right)\beta_{nonpoor}+{\bar{X}}_{poor}\left(\beta_{nonpoor}-\beta_{poor}\right)$$

Hence, in this decomposition approach, the gap in outcome in question is decomposed into (*i*) differences in the means of the determinants between the two groups (endowment/explained component: the first part of the right side of equations 6) and (*ii*) differences in *βs* between the two groups (coefficient/unexplained component: the second part of the right side of equations 6).^2^

## Results

The data of 5188 participants in the first phase of ShECS were used in analysis. The mean (SD) presenting visual acuity was 0.09 (0.21) LogMar (range: -0.3–3). Number (%) of individuals with VI (LogMar > 0.3) was 344 (6.6). The demographic characteristics of the participants are presented in Table 1. As shown, the participants were mostly middle-aged with secondary level education and a body mass index indicating overweight. The majority of them were female, married, employed, and had a type of medical insurance and a middle economic status (third quintile). The proportion of participants with diabetes, hypertension, and cigarette smoking was remarkable. The sum of individuals by variables of cigarette smoking and having a type of medical insurance was not 5188 as a result of some missing values. It must be mentioned economic quintiles were constructed by dividing the economic score obtained from PCA into 5 groups via the “xtile” command in Stata. Eight persons did not answer the questions on asset variables. So, the economic score and economic quintile was unknown for them.

**Table 1:** Demographic characteristics of participants in ShECS, Shahroud, Iran, 2009

**Variables**	**Number**	**Mean**	**SD **
Age (y)	5188	50.93	6.27
Education (y)	4732	7.30	4.67
Body Mass Index (kg/m^2^)	5188	28.40	4.89
Gender (female)	3038	0.5856	0.4927
Marital status			
Married	4794	0.9241	0.2649
Single	67	0.0129	0.1129
Widowed	291	0.0561	0.2301
Divorced	36	0.0069	0.0830
Employment status (unemployed)	98	0.0189	0.1361
Diabetes (yes)	637	0.1228	0.3282
Hypertension (yes)	1982	0.3820	0.4859
Cigarette smoking (yes)	651	0.1256	0.3315
Having a type of medical insurance (yes)	4803	0.9445	0.2289
Economic quintiles			
First	1120	0.2162	0.4117
Second	966	0.1865	0.3895
Third	1983	0.3828	0.4861
Fourth	565	0.1091	0.3118
Fifth	546	0.1054	0.3071
Having home assets (only “Yes” category is presented)
Car	3250	0.6272	0.4836
Motorcycle	1586	0.3061	0.4609
Television	5160	0.9958	0.0650
Bathroom	5163	0.9963	0.0604
Vacuum cleaner	4996	0.9641	0.1860
Washing machine	4621	0.8917	0.3107
Refrigerator	5168	0.9973	0.0519
Computer	3208	0.6191	0.4857
Telephone	5125	0.9890	0.1043
Microwave	427	0.0911	0.2878
Dishwasher	300	0.0579	0.2336

Abbreviation‏: ShECS, Shahroud Eye Cohort Study.

The concentration index of PVA was -0.2451 (95% CI: -0.2783, -0.2119), indicating that PVA did not have an equal economical distribution in the study population. In other words, persons with higher scores on presenting vision (poorer visual acuity) were concentrated among people with a lower economic status. The mean PVA and the prevalence of VI by quintiles of economic status are presented in Table 2. As shown, the mean PVA and the prevalence of VI were the highest in the first quintile (the group with the lowest economic score) with a significant decreasing trend from the first quintile to the fifth quintile (the group with the highest economic score).

**Table 2:** Description of PVA and VI by economic quintiles in Shahroud, Iran, 2009

**Variables**		**Economic Quintiles**					***P*** ** Value**
		**First**	**Second**	**Third**	**Fourth**	**Fifth**	
PVA	Mean	0.161	0.095	0.079	0.053	0.045	< .001^a^
	95% CI	(0.143-0.178)	(0.084-0.106)	(0.071-0.088)	(0.041-0.065)	(0.034-0.056)	
VI	Prevalence	11.96	6.21	5.70	3.36	2.93	< .001^b^
	95% CI	(10.06-13.88)	(4.69-7.74)	(4.68-6.72)	(1.87-4.85)	(1.51-4.35)	

Abbreviations: PVA‏, presenting vision acuity; VI, visual impairment; CI, concentration index. ^a^ One-Way ANOVA (contrast: Polynomial‏ with linear degree‏). ^b^‏ Chi-square test for trend‏.

Before conducting decompositions, we identified the determinants of PVA using a linear regression model. Forward strategy, recommended by Hosmer and Lemeshow,^25^ was used for building the model. According to this strategy, we initially run a simple (univariate) linear regression for each of variables of age, gender, education, marital status, employment status, diabetes, blood hypertension, body mass index, cigarette smoking, having a type of medical insurance and economic status separately. We entered all variables with a significance level below 0.2 in the previous step in a multivariable linear regression. The variables with a significance level below 0.1 in this step including age, gender, education, marital status, employment status, diabetes, and economic status remained in the final model, as presented in Table 3. It should be noted that for the variable of marital status among the levels of married, widowed, and divorced in comparison to single as the reference group, we observed a significant effect only for the married group in univariate analysis. So, we converted it to a binary variable (married/unmarried) in multivariable analysis.

**Table 3:** Decomposing concentration index of PVA in Shahroud, Iran, 2009

**Variables**	***Βeta***	**Mean**	**Elasticity**	**CI**	**Absolute Contribution**	**% Contribution to CI** ^a^
Age (y)	0.0060	50.93	3.2746	-0.0114	-0.0373	15.23
Education (y)	-0.0072	7.30	-0.5635	0.1788	-0.1008	41.11
Gender (female)	0.0200	0.59	0.1257	-0.0673	-0.0085	3.45
Marital status (married)	-0.0378	0.92	-0.3742	0.0258	-0.0097	3.94
Employment status (unemployed)	0.0733	0.02	0.0148	-0.2814	-0.0042	1.70
Diabetes (yes)	0.0361	0.12	0.0475	-0.0517	-0.0025	1.00
Economic quintiles						
First	Reference	-	-	-	-	-
Second	-0.0349	0.19	-0.0697	-0.3811	0.0266	-10.84
Third	-0.0317	0.38	-0.1299	0.1882	-0.0244	9.97
Fourth	-0.0432	0.11	-0.0504	0.6801	-0.0343	13.99
Fifth	-0.0289	0.11	-0.0326	0.8946	-0.0292	11.89
Sum					-0.0613	25.01
Total observed					-0.2243	91.51
Residual					-0.0208	8.49
Total					-0.2451	100

Abbreviations: PVA‏, presenting vision acuity; CI, concentration index. ^a^ Concentration index in PVA (-0.2451).

The concentration index decomposition results are presented in Table 3. Economic inequality in each PVA determinant is shown in the fourth column by their concentration index. For example, the concentration index of age was negative, meaning that older individuals were concentrated among people with a lower economic status. On the other hand, the concentration index of education was positive, indicating that individuals with higher education were concentrated among people with a higher economic status. The contribution of each determinant to economic inequality in PVA is presented in the last column. Education was responsible for 41.11% of the total economic inequality‏ in presenting visual acuity. Economic status (25.01%) and age (15.23%) were the next two important contributors. Other determinants had minor contributions. Among three variables with the highest contribution, age had a much lower concentration index but a relatively high contribution. The reason was having a higher elasticity in comparison with the other two variables, indicating that age participated in PVA inequality predominately via its effect on the outcome variable (PVA).

The total sum of the determinants’ absolute contributions (ie, the deterministic component) was -0.2243, which means the deterministic component explained 91.51% of the total economic inequality‏ in PVA (-0.2243/-0.2451). Therefore, absolute contribution for residual or unexplained component was -0.0208 [(-0.2451)–(-0.2243)]. In other words, 8.49% of the total economic inequality‏ in PVA was not explained by the studied variables, which is due to unmeasured factors.

As mentioned in the Methods section, we divided the sample into two groups by the median of the economic score before the Blinder-Oaxaca decomposition. According to the results, 2387 people (46.08%) had an economic score equal or more than the median (high economic group) and 2793 people (53.92%) had an economic score below the median (low economic group). We compared determinants of PVA between two groups. The results are presented in Table 4. As shown, people in the low economic group were significantly older and had lower education as compared with their counterparts. The frequency of females and people with unemployment and diabetes was also significantly higher in the low versus high economic group. Inversely, there were significantly more married people in the high versus the low economic group. In terms of economic status, quintiles with the highest frequency were the first and third in low and high economic groups, respectively. Dividing people into two groups by the median of the economic score led to zero frequency for some economic quintiles.

**Table 4:** Demographic Differences Between People in High^a^ and Low^b^ Economic Groups, Shahroud, Iran, 2009

**Variables**	**High Economic Group**		**Low Economic Group**		***P Value***
	**Mean/Number**	**SD/Proportion**	**Mean/Number**	**SD/Proportion**	
Age (y)	49.85	5.77	51.85	6.53	< .001^c^
Education (y)	9.53	4.31	5.29	4.00	< .001^c^
Gender (female)	1261	52.83	1773	63.48	< .001^d^
Marital status (married)	2293	96.06	2494	89.29	< .001^d^
Employment status (unemployed)	24	1.01	73	2.61	< .001^d^
Diabetes (yes)	268	11.23	369	13.22	.030^d^
Economic quintiles					
First	0	0.00	1120	40.10	< .001^d^
Second	0	0.00	966	34.59	
Third	1276	53.46	707	25.31	
Fourth	565	23.67	0	0.00	
Fifth	546	22.87	0	0.00	

^a^ High economic group: group with economic score equal or more than median. ^b^ Low economic group: group with economic score lower than median. ^c^ Independent *t* test. ^d^ Chi-squared test.

The Blinder-Oaxaca decomposition results are presented in Table 5. In first part of this table, the results of regression analysis in high and low economic groups are presented. The PVA gap between groups with a high and low economic status was 0.0705 and in favor of the group with a high economic status. Moreover, 87.94% (-0.062/-0.0705) of this gap could be attributed to differences in the determinants between the two groups (explained/endowment component). It means if those in the low economic status group were similar to those in the high economic status group in terms of the studied determinants, the PVA gap between the two groups reached -0.0085. In this component, the calculated differences for all variables were negative (pro-rich inequalities). The most important contributors in this part were education, economic status, and age with sharing 49.35%, 21.77% and 18.39% of the subtotal gap in the explained part, respectively.

**Table 5:** Blinder–Oaxaca Decomposition of PVA Gap Between High^a^ and Low^b^ Economic Groups in Shahroud, Iran, 2009

**Regression Results in High and Low Economic Groups**					
**Variables**	**High Economic Group**		**Low Economic Group**		
	**Coefficient**	***P*** ** Value**	**Coefficient**	***P*** ** Value**	
Age (y)	0.0057	< .001	0.0062	.000	
Education (y)	-0.0054	< .001	-0.0083	< .001	
Gender (female)	0.0314	< .001	0.0086	.381	
Marital status (married)	-0.0347	.116	-0.0397	.065	
Employment status (unemployed)	-0.0064	.771	0.0961	.096	
Diabetes (yes)	0.0279	.006	0.0427	.005	
Economic quintiles					
First	(Omitted)	-	Reference	-	
Second	(Omitted)	-	-0.0343	.001	
Third	0.0016	.833	-0.0140	.290	
Fourth	Reference	-	(Omitted)	-	
Fifth	0.0086	.303	(Omitted)	-	
Blinder-Oaxaca decomposition results	**Prediction**	**95% CI**		***P Value***	
Mean of PVA in high economic group	0.0526	0.0456	0.0596	< .001	
Mean of PVA in low economic group	0.1231	0.1134	0.1327	< .001	
Difference	-0.0705	-0.0822	-0.0588	< .001	
1) Due to Endowment (Explained):	**Prediction**	**95% CI**		***P*** ** Value**	**% Of Total Gap** ^c^
Age (y)	-0.0114	-0.0144	-0.0084	< .001	16.17
Education (y)	-0.0022	-0.0036	-0.0009	.001	3.12
Gender (female)	-0.0306	-0.0378	-0.0234	< .001	43.40
Marital status (married)	-0.0026	-0.0050	-0.0003	.029	3.69
Employment status (unemployed)	-0.0010	-0.0023	0.0003	.119	1.42
Diabetes (yes)	-0.0006	-0.0013	0.0001	.112	0.85
Economic status	-0.0135	-0.0239	-0.0031	.011	19.15
Sub Total Gap (explained part)	-0.0620	-0.0723	-0.0517	< .001	87.94
2) Due to Coefficients (Unexplained)	**Prediction**	**95% CI**		***P *** **Value**	**% Of Total Gap**
Age (y)	-0.0241	-0.1094	0.0611	.578	34.18
Education (y)	0.0134	0.0004	0.0264	.044	-19.01
Gender (female)	0.0226	0.0004	0.0448	.046	-32.06
Marital status (married)	0.0047	-0.0529	0.0624	.871	-6.67
Employment status (unemployed)	-0.0013	-0.0030	0.0004	.127	1.84
Diabetes (yes)	-0.0018	-0.0060	0.0025	.412	2.55
Economic status	0.0202	0.0054	0.0350	.008	-28.65
Constant	-0.0421	-0.1557	0.0714	.466	59.72
Sub Total Gap (unexplained part)	-0.0085	-0.0149	-0.0020	.010	12.06

Abbreviations: PVA, presenting vision acuity; CI, concentration index‏. ^a^ High economic group: group with economic score equal or more than median. ^b^ Low economic group: group with economic score lower than median. ^c^ It was calculated via dividing prediction into total gap (-0.0705) for each variable.

In addition, 0.0085 of the total gap of 0.0705 (12.06%) was due to the differences between the two groups in determinants coefficients (*β*s) and differences in unstudied factors. There was a negative gap (pro-rich inequality) for age, employment status, and diabetes, and a positive gap (pro-poor inequality) for other variables in this part. However, coefficients differences were not statistically significant in this part except for education, gender and economic status.

Figure shows the sorted estimates of contributions for each determinant using the concentration index decomposition and Blinder-Oaxaca decomposition. Contributions in Oaxaca-decomposition were computed via dividing the predictions in the explained part by the total gap (-0.0705). According to Figure, the most important contributors of economic inequality in PVA were the same in two approaches.

**Figure:**
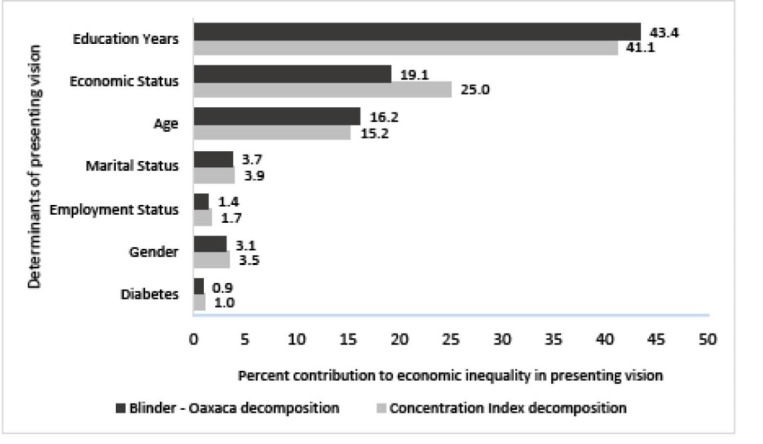
Comparison of Contributions Priority of Determinants to Economic Inequality in Presenting Visual Acuity by Blinder-Oaxaca and Concentration Index Decomposition Approaches in Shahroud, Iran, 2009.

## Discussion

Economic inequality in PVA was estimated and decomposed using two different approaches in this study. The general findings were the same in both approaches; PVA did not have an equal distribution in the study population. It was more concentrated in people with a lower economic status. This finding is in line with the results of many other studies.^26-30^

The contribution of all determinants was positive in both approaches, indicating that all determinants were pro-rich contributors and increased the economic inequality in PVA disfavoring the low economic group.

Education was the factor with the‏ greatest contribution to the PVA economic inequality in both concentration index decomposition and Blinder-Oaxaca decomposition. It was responsible for more than 40% of the inequality observed in PVA. Since the education concentration index was positive and the mean number of successful education years was higher in the high economic group as compared to the low economic group, it could be stated that individuals with more education years were concentrated among people with a higher economic status. On the other hand, a negative regression coefficient for this variable suggests its protective effect on PVA. So, educational status inequality as the most important contributor in this study increased the PVA inequality in favor of the group with a high economic status. In some other studies, this factor has also been reported to be the most important contributor to economic inequality in VI, presenting near vision acuity, and eye care utilization.^9,31,32^ This can be explained by the fact that educated people have higher awareness about benefits of periodic medical eye care and help to maintain their visual acuity in a desired level via timely preventive and therapeutic services.^33,34^ Zheng et al^12^ also stated that a low education level was an independent risk factor for VI. They recommended education promotion interventions in the community level as a way for reducing socio-economic inequalities in presenting vision. Therefore, it can be concluded that one of the most important steps to reduce economic inequality in PVA is to regulate policies with the aim of increasing literacy in people with a low economic status.

Economic status was the second most important contributor to inequality in both approaches. In addition to its indirect effects, economic status can influence health-related problems directly via its effects on using preventive and therapeutic services. People with a better economic status spend more on their health and suffer fewer health problems.^35^ So, improving the economic status of the poorer people and increasing their financial strength to pay for disease prevention, health maintenance and health restoration in disease conditions may result in decreased economic inequalities in health, particularly in PVA.

Age as the third most important contributor to economic inequality in PVA in both approaches had a negative concentration index and its mean was significantly lower in the group with a higher economic status than the group with a lower economic status. So, it could be concluded that in the study population, older people were concentrated in groups with low economic status. On the other hand, age had a direct effect on PVA in regression analysis similar to other studies.^34-36^ So, it can be concluded that age increased PVA inequality in favor of the groups with a high economic status. Thus, welfare programs for enhancing the economic status of older persons can decrease the slope of inequality in age distribution and help to reduce PVA inequality. Khedmati et al^37^ suggested allocating more financial protection to older people in targeted subsidy plans as one of the effective options in this state.

In unexplained components of Blinder-Oaxaca decomposition, the most important variables were education and economic status. Positive values for these variables showed the gap in their effects on PVA was in favor of the low economic group. Regarding their protective role in PVA, it can be stated that the plans with the aim of promoting literacy and economic status in the low economic group can help to reduce inequality via influencing the low economic group more than the high economic group in addition to equalizing literacy and wealth distribution between the two groups.

Another important point was the different sign of values for education, gender, marital status, and economic status in explained and unexplained components, which means education for example was in favor of the high economic group and low economic group for explained and unexplained components, respectively. In other words, although educated people were significantly more concentrated in the high economic group than the low economic group, it had a more protective effect on PVA in the low economic group than the high economic group. We can consider this issue as a privilege for Blinder-Oaxaca decomposition versus concentration index decomposition. It means that Blinder-Oaxaca decomposition shows how a determinant influences the outcome variable separately in groups with different statuses of the living standard variable (high economic/low economic). In concentration index decomposition, a regression coefficient – *βeta* – is estimated for each determinant in total so it is not possible to observe the effects of that determinant on the outcome separately in groups with different levels of the living standard variable in question.

In both decomposition approaches, adding other important factors to the study could increase useful information to help decrease inequality. According to a previous study, 5.7% of the participants had unmet visual acuity needs.^38^ Therefore, access to medical eye care services can be considered as an important factor in these decomposition approaches in order to increase the contribution of deterministic or explained components. Hence, we suggest the role of this factor and other related factors be assessed in future studies.

As stated, the main results were similar in both decomposition approaches. However, it is important to pay attention to some points while comparing these approaches:

First, despite the fact that both approaches are regression-based, they are completely different in terms of methodology. Concentration index decomposition considers the entire distribution of the living standard variable in the measurement and explanation of inequality in the health-related variable while in Blinder-Oaxaca decomposition, the study population is divided into two groups by the living standard variable, and the mean difference of the health outcome variable between the two groups is decomposed as the inequality index. It means Blinder-Oaxaca decomposition considers an inevitably more limited spectrum of the living standard variable in decomposition of inequality in comparison with concentration index decomposition.^2^

In both decomposition approaches, the primary step after estimating the inequality by appropriate index is to identify the health outcome determinants via a suitable regression model. This primary model is the same in both approaches. Variables in this model are determined via biological justification and statistical significance just like modeling in other situations. In Blinder-Oaxaca decomposition, after identifying the determinants of the health outcome variable, we should assess whether these determinants differ‏ systematically between two groups (poor/ non-poor or high-economic/low-economic). It is tested via running a regression analysis on the health outcome variable using living standard as a dummy variable (poor/non-poor or high-economic/low-economic) included alone and in interaction with all other determinants identified in previous step. Then, it is tested whether the coefficients of the living standard dummy variable and its interactions are simultaneously equal to zero. Rejection of this hypothesis is a necessary prerequisite for Blinder-Oaxaca decomposition.^2^ In concentration index decomposition, on the contrary, it is not obligatory to test any hypotheses as a decomposition precondition.

Concentration index decomposition estimates only one mean and one regression coefficient for each determinant with the assumption that each determinant has a similar effect throughout the distribution of the living standard variable.^22^ Blinder-Oaxaca decomposition, on the other hand, is essentially based on this assumption that the value and effect of each determinant are different between the two groups (poor/non-poor or high-economic/low-economic). So, it estimates the means and regression coefficients of the determinants separately in each group and uses the differences of these means and coefficients between the two groups for decomposition.^2^ Therefore, the health outcome gap between two groups (poor/non-poor or high-economic/low-economic) is decomposed to two components of explained or endowment (differences in means of determinants between two groups) and coefficient or unexplained (differences in regression coefficients between two groups) in Blinder-Oaxaca decomposition. So, in concentration index decomposition, we calculate the total contribution of each determinant to health outcome inequality while in Blinder-Oaxaca decomposition, each determinant contributes to health outcome inequality in two parts. In other words, the total contribution of each determinant is equal to the sum of its contribution in explained and unexplained parts in Blinder-Oaxaca decomposition.

Second, the contribution of unstudied variables, known as the residual component, can be calculated easily in concentration index decomposition while it is not possible to determine their exact contribution in Blinder-Oaxaca decomposition.^2^ This can be considered an important advantage for concentration index decomposition since it can be used to suggest future studies to explain inequality.

Third, as previously mentioned, the main assumption of concentration index decomposition is lack of interaction between the determinants and the variable related to living standard.^2^ Madden^22^ believes this cannot be a true assumption in all situations. In his study on Oaxaca decomposition of low birth weight inequality, he stated that the effects of ill health as a determinant for low birth weight may be different by the income level due to allocation of other resources for offsetting ill health effects by richer families. Therefore, when the effects of the determinants on the outcome are different in terms of the living standard variable in question, it is better to use Blinder-Oaxaca decomposition to explain inequality. This can be important from the public health perspective because it emphasizes specific policies for each group eg, poor vs. non-poor or low economic vs. high economic when the effects of the determinants on the outcome are different between the two groups. Another suitable situation for applying Blinder-Oaxaca decomposition is when the variable in question for measuring the inequality of an outcome has a binary nature in the study population such as race (black/white), location (urban/rural), etc.

Fourth, the standard error of each estimate is easily calculable in Blinder-Oaxaca decomposition, so it is possible to calculate the confidence interval of the contribution of each determinant. In concentration index decomposition, however, the contribution of each factor is calculated via multiplying its concentration index by its elasticity. So, the confidence interval of the contribution is not calculable directly and a bootstrap method is advised.

## Future Research Directions

As the last consideration, it should be mentioned both methods used in this study suffer from some flaws. The concentration index of a health variable will differ by different socio-economic measures if the health variable is associated with changes in an individual’s rank on shifting from one measure to another. We suggest checking concentration index changes using different socio-economic measures in future studies. Blinder-Oaxaca decomposition, on the other hand, is a mean-based decomposition. Therefore, the possible heterogeneity of the distribution of covariates in full distribution of the outcome of interest remains unclear. It can be addressed more deeply via other methods such as Recentered Influence Function (RIF) which estimates the effect of explanatory variables on the unconditional quantiles of an outcome variable^39^ or representing an alternative to quantile regression via focusing counterfactual distributions.^40^ We recommend performing a comparison study on Blinder-Oaxaca decomposition, RIF, and methods introduced by Chernozhukov et al^40^ as methods for exploring the marginal distribution of the outcome of interest in the future.

## Strengths and Limitations

We compared two inequality decomposition approaches and presented some of their benefits and shortcomings. This can be a guide for choosing any of these methods in future studies. However, we had some limitations in this study. We performed decomposition approaches and compared them only on a single data set. We do not know if the results will be the same in other situations and by other data sets. So, we suggest a simulation study for comparing the results of these decomposition approaches using different data. As another limitation, it should be noted this was a cross-sectional study. So it is difficult to have a causal interpretation for observed relationships. Although decomposition approaches determine the contribution of each variable, according to O’Donnell et al,^2^ decomposition as a mathematical tool only provides explanation in a statistical sense in the absence of causal evidence. Since our data were derived from the first phase of a cohort study, we will assess these inequalities longitudinally in the future in hopes of producing useful guides for health policy-makers. In addition, the generalizability of our results may be slightly low as a result of focusing on people aged 40-64 in the city of Shahroud. However, socio-economic characteristics of Shahroud population were approximately similar to them in Iranian urban population on average based on information provided by Statistical Centre of Iran. So, it seems our results can be generalizable at least to the Iranian urban population aged 40-64 years old.

## Conclusion

We estimated the economic inequality of PVA by two indices‏. Both indices showed that individuals with poorer visual acuity were more concentrated among people with a lower economic status. The main drivers of this inequality were education, economic status, and age in decomposition of both inequality indices. Therefore, it can be concluded that setting appropriate interventions to promote literacy and income in people with low economic status, formulating policies to address the economic problems in the elderly, and paying more attention to their vision problems can have an effective role in reducing economic inequality in visual acuity. In addition, assessment of the effect of other factors, including factors related to access to medical eye care, can help to better explain inequality and minimize the share of unexplained factors. The main benefits of concentration index decomposition over Blinder-Oaxaca decomposition are considering the entire distribution of the variable of standard living and exact estimation of the contribution of unmeasured factors. The main superiority of Blinder-Oaxaca decomposition on the other hand is to determine how factors influence the outcome separately in advantageous and disadvantageous groups. It can be important in designing interventions specifically for each group.

## Acknowledgements

ShECS was supported by the Noor Ophthalmology Research Center, Tehran, Iran and Shahroud University of Medical Sciences, Shahroud, Iran. This study was supported by Tehran University of Medical Sciences, Tehran, Iran as a part of PhD thesis.

## Ethical issues

The Ethics Committee of Shahroud University of Medical Sciences, Shahroud, Iran approved this study in accordance with the tenets of the Declaration of Helsinki, and all participants signed written informed consents. The Institutional Review Board code of ShECS is 8737. More details about ethical aspects of this study have been already presented.^15^

## Competing interests

The authors declare that they have no competing interests.

## Authors’ contributions

AM analyzed the data, interpreted the results and prepared the manuscript. MHE planned the original study, reviewed data analysis, and critically revised the manuscript. HZ contributed to the statistically conceptualization of the paper. HH planned the original study, made substantial contribution in data preparation, and reviewed manuscript. AF planned the original study, supervised the study, contributed to the conceptualization of the paper and critically revised the manuscript. All authors read and approved the final manuscript.

## Authors’ affiliations

^1^Department of Epidemiology and Biostatistics, School of Public Health, Tehran University of Medical Sciences, Tehran, Iran. ^2^Ophthalmic Epidemiology Research Center, Shahroud University of Medical Sciences, Shahroud, Iran. ^3^Noor Ophthalmology Research Center, Noor Eye Hospital, Tehran, Iran.

## Key messages

**Implications for policy makers**

- Individuals with higher scores on presenting vision (poorer visual acuity) are concentrated among people with a lower economic status.
- Education and economic status are responsible for more than 60% of the inequality observed in presenting visual acuity.
- Formulating policies aimed at reducing inequality in education and improving the economic status of the poorer people can play an effective role in narrowing the presenting vision inequality.

**Implications for public**

We showed presenting vision acuity (PVA) was unequally distributed among people with different economic statuses. People with lower economic status had more visual impairment. We determined some contributors of this inequality via decomposing the inequality indices. Education and economic status were the main contributors to inequality in presenting visual acuity. Considering the fact that people with higher education have higher awareness about benefits of periodic medical eye care and help to maintain their visual acuity in a desired level via timely preventive and therapeutic services, improvements in education with more emphasis on illiterate and low educated people can be a valuable step towards reducing presenting vision inequality. Furthermore, increasing the financial strength of the poorer people to pay for disease prevention and health maintenance may result in decreased economic inequalities in health, particularly in PVA.
